# Bidirectional selective genotyping approach for the identification of quantitative trait loci controlling earliness *per se* in winter rye (*Secale cereale* L.)

**DOI:** 10.1007/s13353-015-0294-5

**Published:** 2015-06-12

**Authors:** Beata Myśków, Stefan Stojałowski

**Affiliations:** Department of Plant Genetics, Breeding and Biotechnology, West-Pomeranian University of Technology in Szczecin, Słowackiego 17, 71-434 Szczecin, Poland

**Keywords:** BSG, Heading, QTL, Rye

## Abstract

The genes controlling earliness of plants include genes responsible for vernalisation (Vrn) and photoperiod (Ppd), and those that are not entirely associated with a response to temperature or light. The last group of loci is known as earliness *per se* (Eps). Eps genes have been most commonly reported in the scientific literature as quantitative trait loci (QTL). The objective of this study was to use a bidirectional selective genotyping (BSG) method for the identification of loci controlling Eps, assessed at the heading stage in a newly developed rye population of recombinant inbred lines. We identified four linkage groups representing fragments of chromosomes, 1R, 5R, 6R and 7R, and containing, in total, 155 DArT markers significantly associated with earliness in rye. The results are discussed in the context of previously published QTL analyses.

**Electronic supplementary material:**

The online version of this article (doi:10.1007/s13353-015-0294-5) contains supplementary material, which is available to authorized users.

## Introduction

The time of flowering is an important trait that affects plants’ adaptability to environmental conditions. Genes underlying the transition from vegetative to generative phases of growth are grouped into three types: genes responsible for vernalisation (*Vrn*); those controlling reaction to photoperiod (*Ppd*); and genes not exclusively associated with a response to temperature or light (Snape et al. 2001), commonly known as earliness *per se* (Eps) genes. Genes involved in this process may act as the main determinants of time variation in winter or spring crop flowering (Laurie et al. 2004). The characterisation of Eps genes may enable selection of early crop varieties and may also be important because some research suggests that loci controlling Eps may also influence yield and yield-related traits (Lewis et al. 2008). A relationship between the localisation of loci controlling Eps and pre-harvest sprouting and α-amylase activity was also found (Myśków 2012; Myśków et al. 2012).

Eps genes are most commonly reported as quantitative trait loci (QTL) (Law et al. 1998; Buck-Sorlin and Börner 2001; Kikuchi et al. 2009). Their effects are significantly less evident in comparison with genes controlling Ppd or Vrn. Eps genes were located in various genome regions when different crosses were applied to investigate the genetic base of earliness. *Ppd* and *Vrn* were reported to be in the minority but showed strong phenotypic effects (Kato et al. 1999; Laurie et al. 2004).

The development of recombinant inbred lines (RILs) with simultaneous bidirectional selective genotyping (BSG) identifies specific genotypes that represent extreme variant-related traits in each breeding cycle (Gallais et al. 2007). Repeated selection of extreme variants year after year results in the identification of genotypes phenotypically stable in different environments (vegetative seasons). Such selection of genes should increase the prevalence of the trait under investigation and identify possible genetic markers. This approach was introduced by Lebowitz et al. (1987), followed by Lander and Botstein (1989), who proposed the term ‘selective genotyping’ for QTL mapping based on selected groups of progeny. BSG allows for the precise identification of QTL in situations in which phenotyping and genotyping numerous populations is too costly or not feasible (Navabi et al. 2009). Recently, this procedure was successfully applied for analysis of the genetic architecture of α-amylase activity in rye grain (Masojć et al. 2011).

We have previously used QTL-CIM (composite interval mapping) analysis in four different rye RIL populations (marked as K, L, M and S) to find loci controlling Eps (Myśków 2012; Myśków et al. 2012). Earliness was assessed at the heading stage, so it was also named ‘heading earliness’ (HE). The purpose of this study was to apply the BSG approach for the identification of rye genes related to HE and to compare the results of these two methods.

## Materials and methods

Experiments were conducted on the newly developed winter rye RIL population C599 × 620-2, marked as R. The parental inbred line C599 was obtained from DANKO Plant Breeding Ltd. (Choryń, Poland) and reproduced by self-pollination in the West Pomeranian University of Technology for over ten generations before hybridisation. Line 620-2 was developed by M. Łapiński (Agricultural University in Szczecin) in the 70s of the 20th century from the cultivar ‘Dańkowskie Złote.’ These lines differ in terms of earliness by about 3 days (C599 is moderate early, while 620-2 is moderate late). The remaining pairs of parental lines of four mapping populations (K, L, M and S), published previously (Milczarski et al. 2011), were unrelated to C599 and 620-2. They also varied in terms of Eps not extremely. Moderate differences with respect to earliness allowed for obtaining all crossings in the normal field conditions.

A subset of 74 RILs derived from over 1,000 plants of the F_2_(S_1_) generation of the C599 × 620-2 interline cross was obtained after bidirectional selection. Plant material was grown at the field experimental station of the West Pomeranian University of Technology located in Szczecin (Poland). The assessment of earliness in each generation, starting from S_1_ (2006), was conducted using a nine-point scale (Masojć and Milczarski 1999). First, single plants of the S_1_ and the whole S_2_ generation were sowed, followed by 5–8 plants of selected S_3_–S_7_ recombinant lines. From S_3_, the earliness was assessed for all 5–8 plants within each RIL and the mean values of Eps were considered for selection. Grains from one ear representing single plants of each early and late line were chosen for obtaining the next generation.

Two hundred inbred lines representing phenotypic extremes (marginal values on the nine-point scale, which corresponds to 5–15 days in heading time, depending on the year) were obtained in the S_6_ generation. In spring of 2011, the leaves from several plants of each S_6_ line were collected for molecular analysis. After the final phenotypic analysis of the S_6_ generation, 74 lines (38 early lines and 36 late lines) represented by the most extreme phenotypes were selected and subjected to analysis by diversity array technology (DArT).

DNA isolation was performed using the GenElute Plant Genomic DNA Miniprep Kit assay (Sigma-Aldrich, USA). DArT analysis was conducted at Diversity Arrays Technology P/L, Yarralumla, Australia (http://www.diversityarrays.com).

Among all polymorphic DNA markers, segregating markers associated with a studied trait were selected. Analysis based on a Chi-square test was conducted separately for the group of early and late genotypes. Accordance between observed marker segregations (within a group) and the expected distribution was considered as a proof of independence between the marker and the studied trait (for randomly selected RILs, a ratio of 1:1 is expected). A significant deviation from the expected segregation ratio was considered as an indicator that the analysed marker was associated with the earliness. Markers were definitively recognised as associated with earliness QTL if statistically significant differences between observed and expected values (at *P* = 0.05) were reported simultaneously in both studied groups: abundant alleles in early lines were in the minority in late lines and vice versa.

Markers selected by the Chi-square test as being significantly associated with earliness-related genes were then used for the development of genetic linkage maps using the JoinMap 3.0 package (van Ooijen and Voorrips 2001) under a logarithm of odds (LOD) score of at least 4. A linkage group was assigned to a particular chromosome based on the presence of DArTs previously localised on different genetic linkage maps of rye (Milczarski et al. 2011; Myśków 2012). The final localisation of genetic markers was established by running the ‘order’ command according to ‘shared markers’ from consensus rye maps described by Milczarski et al. (2011).

In addition, the Kruskal–Wallis (K–W) test (Kruskal and Wallis 1952) was used to distinguish markers of more general importance within the set of DArTs selected by the Chi-square test in population R. The relationship between markers and the studied trait was assessed also within four genetically unrelated mapping populations (K, L, M and S) using the MapQTL 5.0 package (van Ooijen 2004). Markers significant at *P* = 0.05 were considered as potentially applicable for selection within genetically different germplasms.

Correlation coefficients between earliness in different years of the study were established using the STATISTICA version 9.0 package (http://www.statsoft.com/).

## Results and discussion

Heading time is strongly affected by environmental conditions. The aim of bidirectional selection performed in each year during development of the RIL-R population was to identify phenotypically stable genotypes representing groups of early and late RILs. Comparison of data obtained for the finally selected 74 lines, when earliness of subsequent generations from S_3_ to S_7_ was assessed, revealed high rates of correlation between HE in different years of the study (0.76–0.95). The significance of all correlation coefficients and the highest value obtained by the pair S_6_–S_7_ proved the phenotypic stability of earliness in the selected set of RILs used for genotyping. Based on DArT analysis, 3,566 markers were obtained, of which 2,819 were polymorphic. By applying the Chi-square test, we selected 155 DArT markers associated with a segregation-related trait (see the electronic supplementary material, ESM 1). Values of the Chi-square test for selected markers were significant at *P* = 0.05 to *P* = 0.0001 (3.90 ≤ *χ*^2^ ≤ 23.06).

**Fig. 1:**
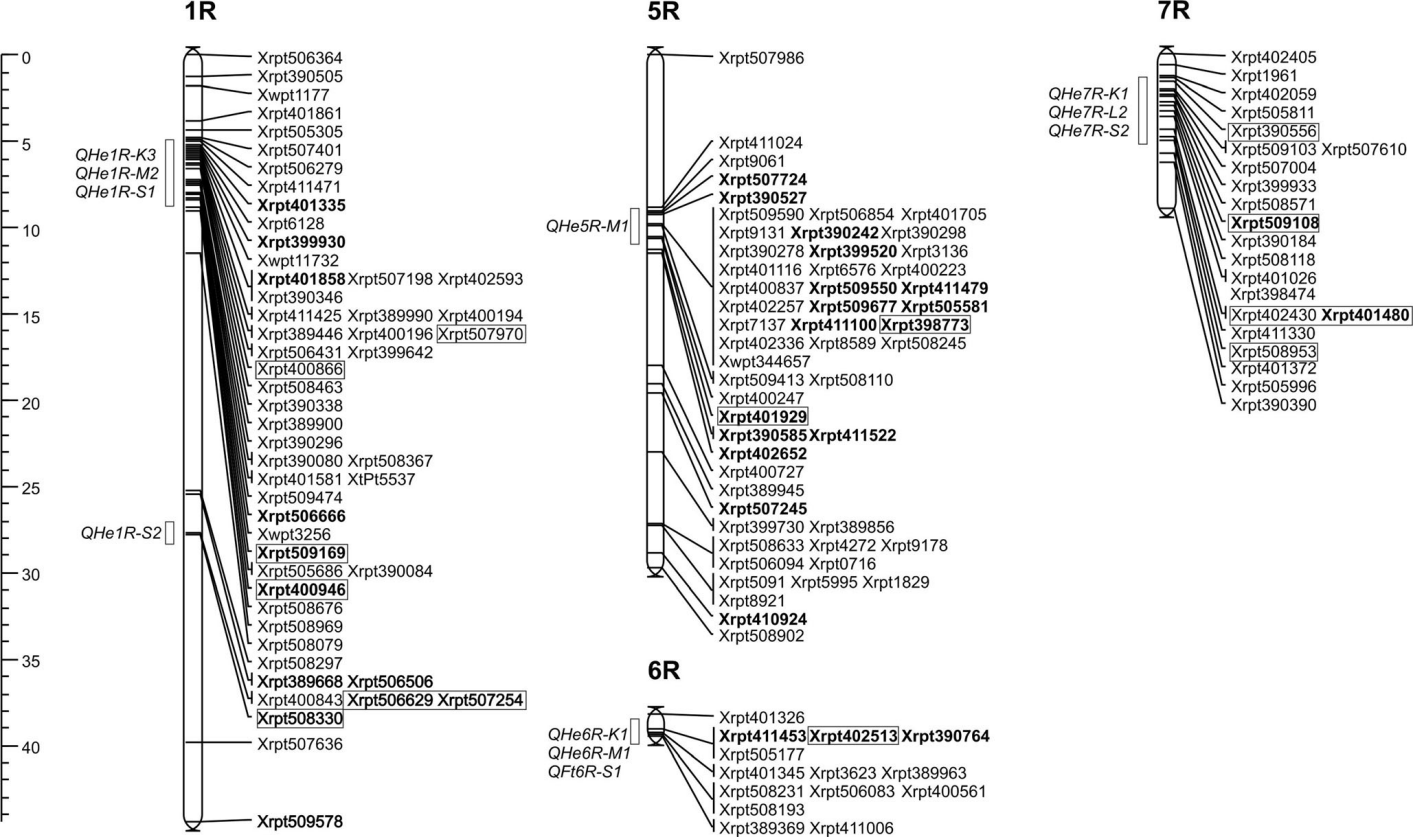
Genetic map of rye chromosomes with loci significant for heading earliness (HE) in population R (C599 × 620-2). Markers significant in four previously published populations (Myśków 2012) are indicated: *in frames*, detected by composite interval mapping (CIM); *in bold*, significant in Kruskal–Wallis (K–W) tests (performed in this study); on the *left*, approximate position of quantitative trait loci (QTL) for HE, determined on the basis of framed markers

After analysis of the 155 selected markers using JoinMap 3.0 software, we obtained four linkage groups (Fig. 1) representing fragments of chromosomes: 1R, 5R, 6R and 7R (comparison of linkage groups of the RIL-R population with the consensus genetic map of rye is shown in the electronic supplementary material, ESM 2). These groups consisted of 52, 53, 14 and 22 marker loci, respectively. The length of chromosomal maps composed of markers for earliness covered 44, 30, 1 and 9 cM distances, respectively (Fig. 1).

Earliness trait-associated loci have been identified across seven rye chromosomes based on CIM of four mapping populations (Myśków 2012). At least 15 QTL were identified on each map. Unfortunately, many QTL detected did not allow the identification of the most beneficial markers for breeding. Results from the BSG approach indicating the presence of earliness genes on four chromosomes cannot explain the genetic background of heading time control in detail. Nevertheless, these results indicate genes that may be of particular interest for the breeding practice, due to their significant and environmentally stable phenotypic effect.

The methodological approach applied in this study allowed the selection of genotypes responsible for different extreme phenotypes continuously manifested in various environmental conditions over the past 5 years of experiments. As was suggested by Navabi et al. (2009), such restrictive selection of plants should allow for the identification of markers closely linked with the loci controlling Eps. However, it should be stressed that the one cross-bred combination analysed in this study does not allow generalisation about all practical aspects.

Genetic markers linked with QTL are said to be universal only on the condition that they are useful in genetically different plant material. In order to verify, at least partially, which markers could be the most applicable, we compared the results of the present study with previous research based on the QTL-CIM analysis of four different rye RIL populations (marked as K, L, M and S) (Myśków 2012; Myśków et al. 2012). Additionally, the results from QTL-CIM analysis for the K, L, M and S populations were verified in this study by the K–W test, and the data were used for the validation of results from the R population (see the electronic supplementary material, ESM 3). There were 33 DArTs detected in the RIL-R population which were indicated as significant by the K–W test in at least one of the four mapping populations (Fig. 1, ESM 3). Thus, a limited number of markers revealed a relationship with Eps when genetically different rye inbred lines were analysed.

The results presented in this study suggest that most of the genome regions indicated in the R population with the use of markers selected after applying the Chi-square test were consistent with those previously identified as QTL for HE (Fig. 1, Table 1).

**Table 1:** List of markers significant for earliness *per se* (Eps) in population R (hybrid C599 × 620-2) previously mapped in other populations (Myśków 2012) within intervals of quantitative trait loci (QTL) for heading earliness (HE)

Marker	Position		*χ* ^2^ in group of:				QTL for HE in which the marker was present (Myśków 2012)	
	Chromosome	cM	Early RILs		Late RILs		Symbol	Population
XrPt507970	1R	2.031	6.12	*	10.31	**	*QHe1R*-*K3*	K
XrPt400866	1R	2.238	10.80	**	4.50	*	*QHe1R*-*S1*	S
XrPt509169	1R	4.277	7.53	**	4.83	*	*QHe1R*-*M2*	M
XrPt400946	1R	4.586	7.53	**	5.44	*		
XrPt506629	1R	23.925	4.24	*	4.00	*	*QHe1R*-*S2*	S
XrPt507254								
XrPt508330	1R	23.992	5.12	*	4.00	*		
XrPt508501	1R	Unmapped	5.76	*	4.24	*	*QHe1R*-*M3*	M
XrPt398773	5R	9.843	23.06	***	9.00	**	*QHe5R*-*M1*	M
XrPt401929	5R	10.594	19.2	***	5.45	*	*QHe5R*-*M1*	M
XrPt402513	6R	0.827	4.50	*	5.76	*	*QHe6R*-*K1*	K
XrPt390556	7R	1.562	5.76	*	10.31	**	*QHe7R*-*S2*	S
XrPt509108	7R	2.713	4.24	*	4.00	*	*QHe7R*-*K1*/ *QHe7R*-*S2*	K, S
XrPt401480	7R	4.310	4.24	*	8.26	**	*QHe7R*-*K1*/ *QHe7R*-*S2*	K, S
XrPt402430							*QHe7R*-*S2*	S
XrPt508953	7R	4.963	4.24	*	10.31	**	*QHe7R*-*S2*	S

*Significant at *P* < 0.05

**Significant at *P* < 0.01

***Significant at *P* < 0.001

The map of chromosome 1R for the R population contains a region-length at the 44 cM distance (Fig. 1). In this interval, we identified the same group of markers that was previously located in QTL for earliness on three different crossbreed maps (K, M, S). Moreover, an additional group of markers that mapped in QTL *QHe1R*-*S2* (Fig. 1) was detected only in the S population (Myśków 2012).

In the first group of homologous chromosomes of cereals related to rye, i.e. wheat and barley, earliness loci were also detected. Among them, genes controlling photoperiod as well as those not associated with temperature or light were identified (Law et al. 1998; Buck-Sorlin and Börner 2001; Kikuchi et al. 2009), including the *EpsA*^*m*^*1* from diploid wheat, which has been studied in detail (Bullrich et al. 2002; Valárik et al. 2006; Lewis et al. 2008; Faricelli et al. 2010).

The 5R linkage group was constructed with 53 markers distributed over the 30 cM distance (Fig. 1), where some bins with markers in complete linkage have been found. The most numerous bin contained 19 DArTs. It was localised in a region where there was a previously identified QTL for earliness detected in the M population (Myśków 2012; Myśków et al. 2012). So far, QTL analysis has suggested the presence of single QTL detected on the 5R chromosome in three different rye crossbreeds (Masojć and Milczarski 1999; Myśków 2012). It is not clear whether the identified regions are homologous due to insufficient precision in the localisation of QTL intervals or the lack of common markers.

It is highly probable that the Eps gene/genes detected on the 5R chromosome within the R population is identical to the QTL found on the previously analysed map of the M population (Myśków 2012). The likely identified interval is also homologous to the region carrying genes responsible for vernalisation, *Vrn*-*R1* (Efremova et al. 2006) (also known as *Sp1*; Plaschke et al. 1993). The same locus, named *Hd2*, may also have been detected in the L population (Masojć and Milczarski 1999). Therefore, one could conclude that earliness QTL established for vernalisation sensitivity genes of non-differentiated populations (experiments conducted on winter crops) correspond to the location of these genes. The presence of QTL positions that correspond to the main gene is not an unusual phenomenon. Some QTL detected in one population are identified as loci of main genes in other germplasm (Huang et al. 1996; Börner et al. 2000; Myśków et al. 2014; Święcka et al. 2014).

The smallest and the shortest group from the 6R chromosome, composed of 14 completely linked loci (Fig. 1), is homologous to the region where earliness QTL on three rye maps were previously detected: *QHe6R*-*K1*, *QHe6R*-*M* and *QFt6R*-*S1* (Myśków 2012). To date, one to three earliness QTL have been identified on chromosome 6R using different biological materials (Stojałowski and Łapiński 2002; Myśków 2012).

The presence of an earliness QTL on chromosome 7R was reported for the first time by Börner et al. (2000). It was extended over the 97 cM distance (major part of the chromosome), which suggests the possible occurrence of more than one locus involved in the control of this trait within the identified interval. It is possible that the small number of markers on the genetic map described by Börner et al. (2000) does not allow for the differentiation of QTL. Later studies (Myśków 2012) identified two to three Eps QTL on chromosome 7R, depending on the mapping population. The region essential for earliness-related markers in the R population confirmed the location of QTL identified on maps from three different mapping populations (Fig. 1).

Comparing to CIM, the BSG method applied in this work, together with statistical analysis based on the Chi-square test, seems to be insufficient to detect all QTLs. QTLs revealing small and unstable phenotypic effects remain undetected, but it is possible to indicate genomic regions with the highest significance for earliness. Based on the results of this study, markers from 5R seem to be the most influential for earliness heading control in the R population (ESM 1).

A total Chi-square value (sum of *χ*^2^ values of the early lines and late lines groups) above 20 was attributed to 51 markers from 5R and one marker from 1R. When the total value of Chi-square was between 10 and 20 (10 < *χ*^2^ ≤ 20), we found 44 markers from 1R, 18 from 7R, six from 5R and one from 6R. Most of the DArTs from 6R were characterised by a total *χ*^2^ ≤ 10, suggesting a less significant effect of these genes.

Studies of the R population have identified previously unknown novel markers for earliness heading. Among 155 DArT markers, 94 were detected for the first time.

Compared with BSG analysis, QTL based on genetic maps provided more insight into quantitative trait assessment (parameters like: LOD, logarithm of odds; a, the additive allele effect; R^2^, percent of explained variance) across genes; however, these values vary depending on the environmental conditions and plant material under investigation. QTL found to be significant (high LOD) on one population map may explain most of the observed variance (high R^2^), although they may not be detectable or may be less significant on another population map.

Rye is usually grown on light, sandy soils where drought occurs frequently. Early genotypes can partly avoid droughts as they use the water stock accumulated during winter and early spring more effectively. Earliness in rye is controlled by multiple genes and their accumulation is necessary for obtaining early genotypes. This process can be facilitated by marker-assisted selection (MAS). From a practical point of view, to fulfil MAS needs, it is necessary to identify more common QTL alleles in various genetic materials. By applying QTL analysis together with comprehensive genetic maps and/or BSG in several populations of different origins, an effect similar to association mapping can be obtained.

## Conclusions

The results presented in this paper show the potential of mapping populations characterised by extreme trait-related groups to detect fewer loci. The detected quantitative trait loci (QTL) seem to be more repeatable and, therefore, more reliable. QTL on chromosome 5R was the most efficient for earliness *per se* (Eps) control in the RIL-R population, but those on 1R, 6R and 7R chromosomes seem to be more commonly distributed within rye germplasm, because each of them was detected in four different biparental mapping populations.

## Electronic supplementary material

Below are the links to the electronic supplementary material.

ESM 1 (XLS 62 kb)

ESM 2 (PDF 136 kb)

ESM 3 (XLS 51 kb)
