# Supplementary material for: Bidirectional selective genotyping approach for the identification of quantitative trait loci controlling earliness per se in winter rye (Secale cereale L.)
Source: J Appl Genet. 2015 Jun 12;57:45–50. doi: 10.1007/s13353-015-0294-5 (PMC4731430; doi:10.1007/s13353-015-0294-5)

ESM2 - electronic supplementary material

Comparative mapping of linkage groups constructed for RIL-R population (with the use of DArT markers revealing significant associacion with Eps genes) and consensus genetic map of RIL-M population (Milczarski et al. 2011)

Markers common for both mapping populations are printed ***in red****.*

Chromosome 1R

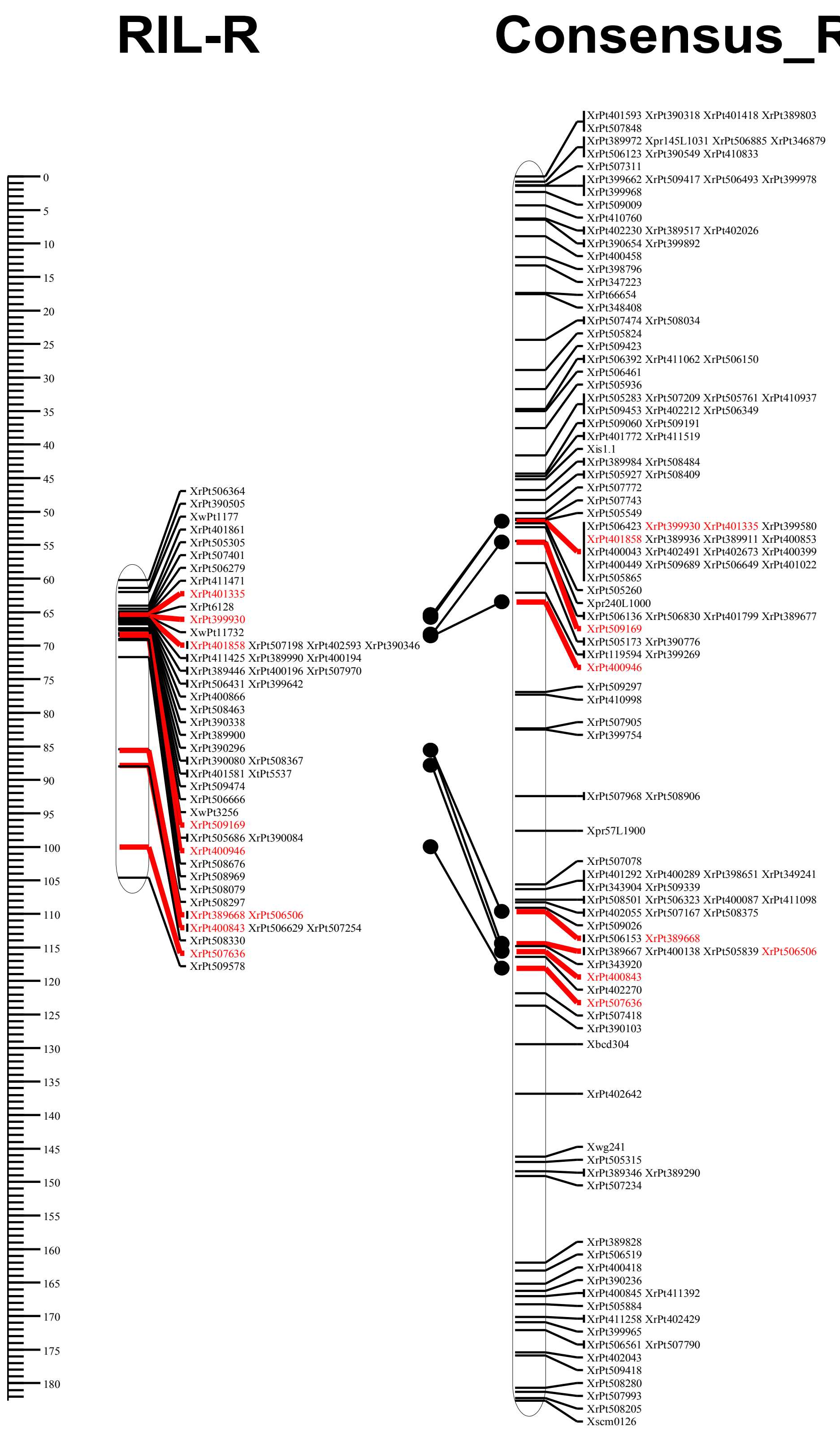

Chromosome 5R

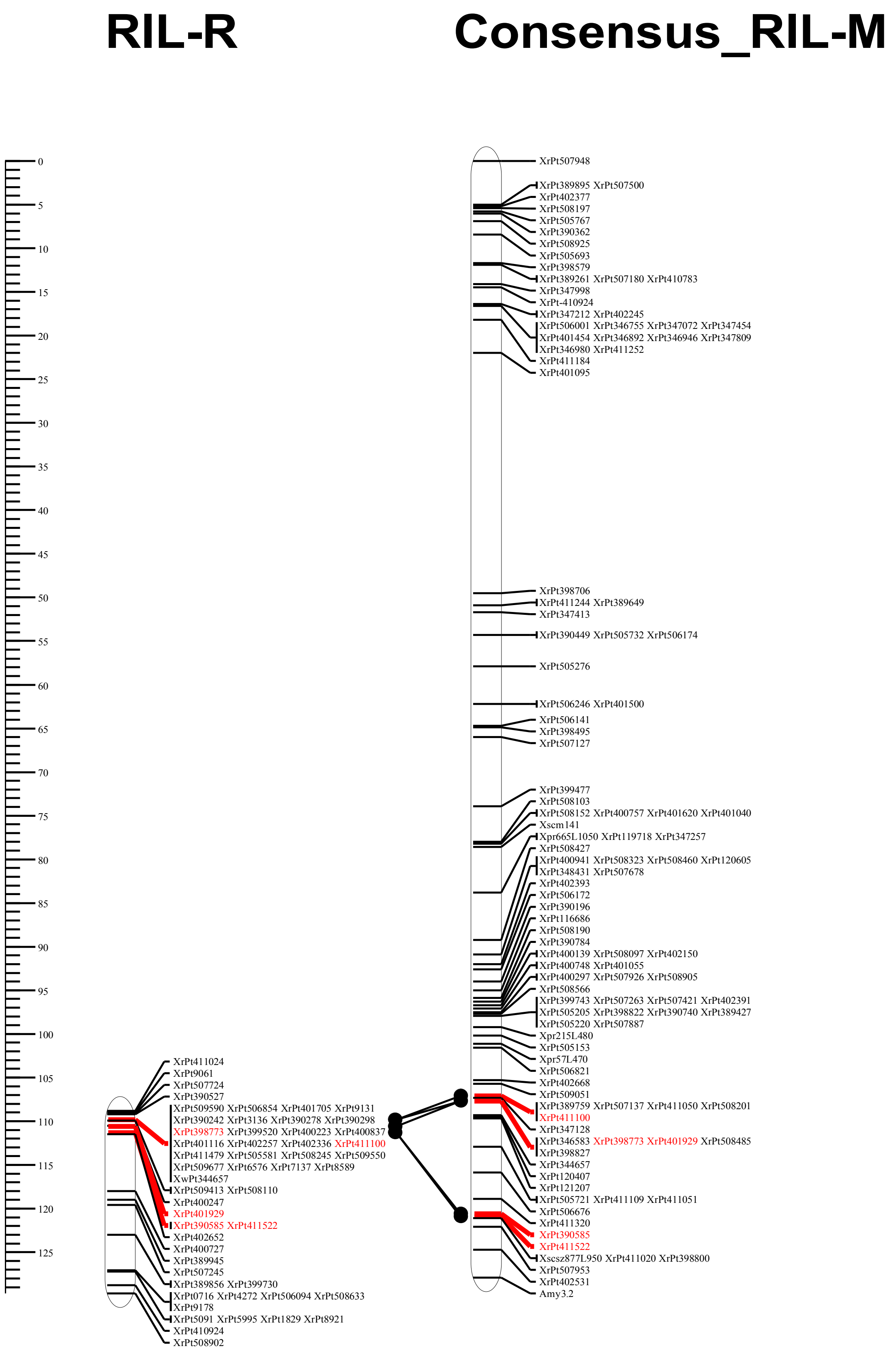

Chromosome 6R

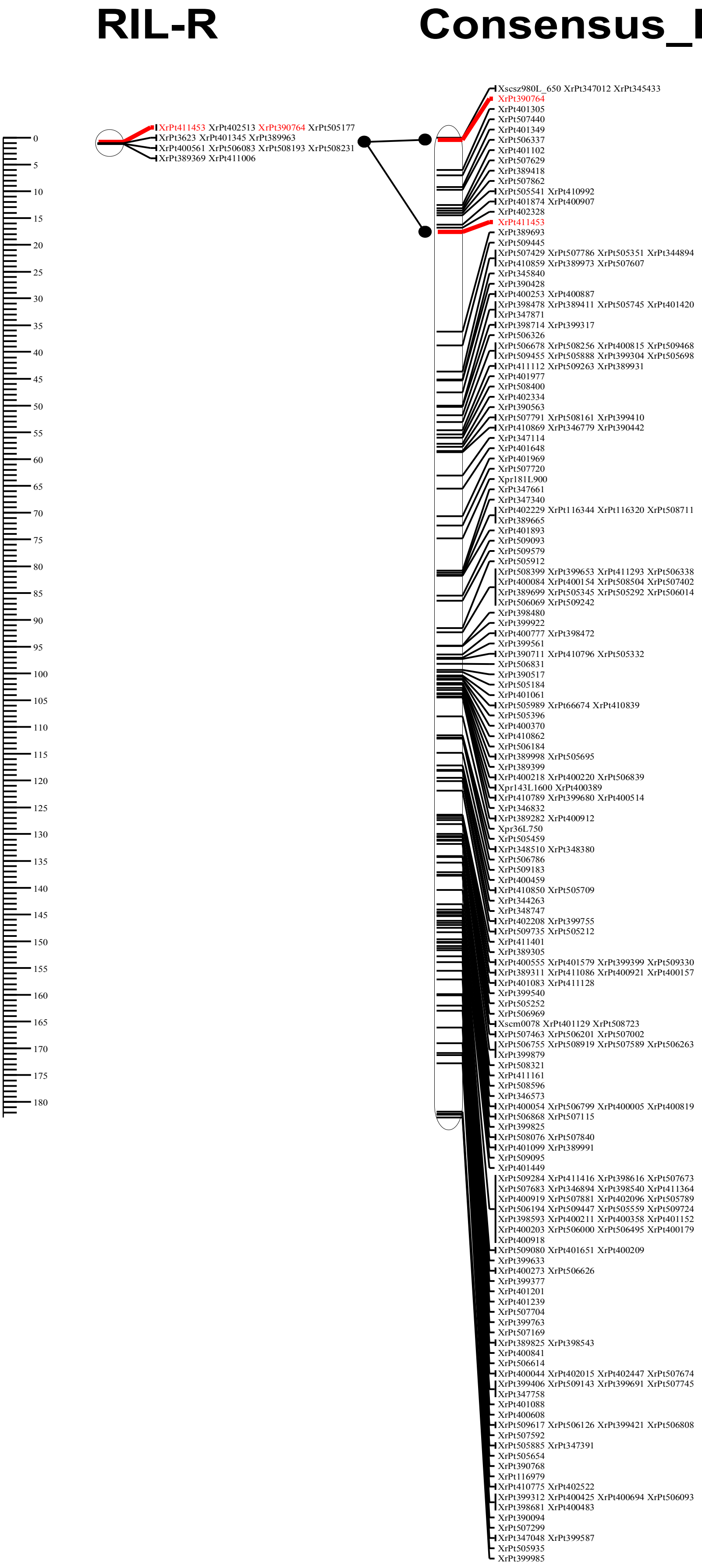

Chromosome 7R

RIL-R Consensus_RIL-M

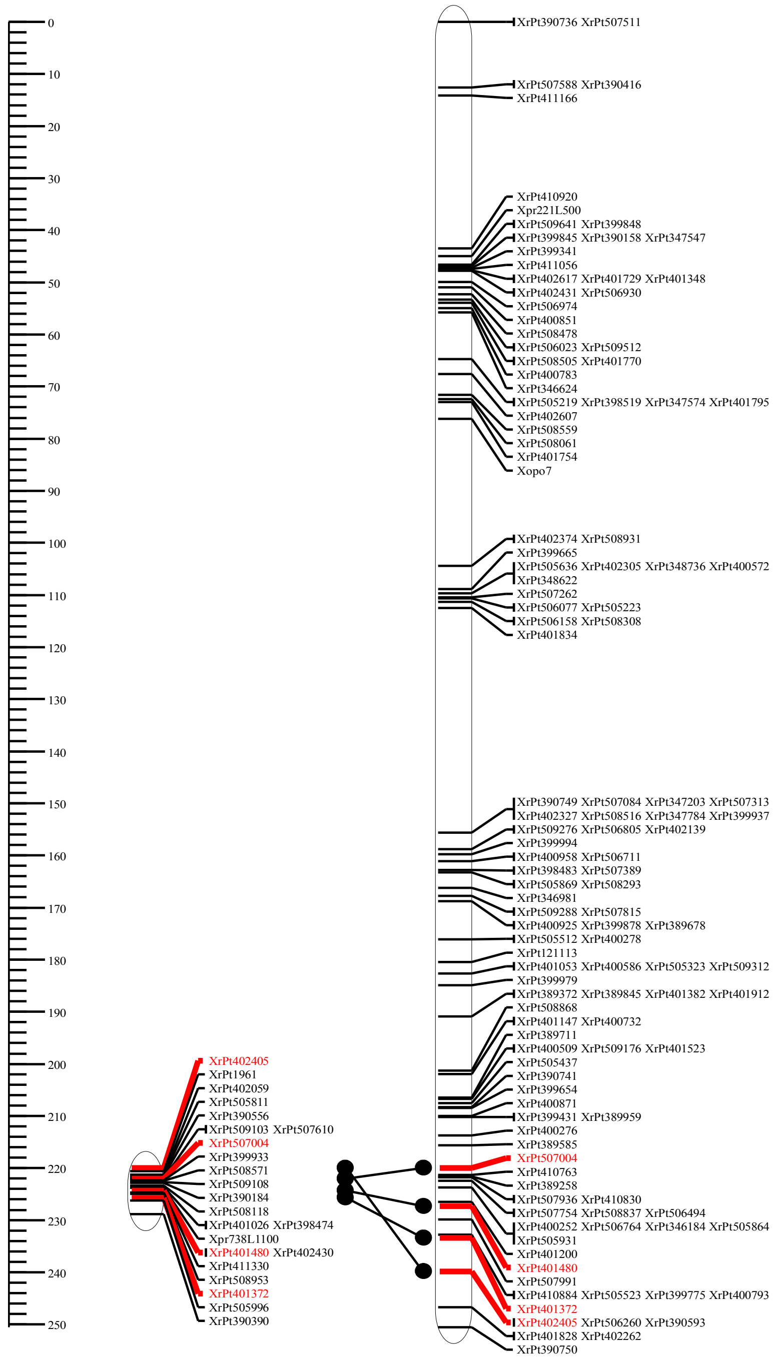

Supplement: Supplementary file 2 — (PDF 136 kb) [file 13353_2015_294_MOESM2_ESM.pdf]
